# Effect of progesterone levels on the hCG trigger day and the progesterone-to-basal progesterone ratio on pregnancy outcomes in fresh IVF/ICSI cycles with GnRH antagonist protocol: a retrospective cohort study

**DOI:** 10.3389/fendo.2025.1653555

**Published:** 2025-12-17

**Authors:** Lu Guan, Haicui Wu, Yuan Li, Qihui Liang, Xina Zhen, Shan Xiang, Fang Lian

**Affiliations:** 1 First College of Clinical Medicine, Shandong University of Traditional Chinese Medicine, Jinan, Shandong, China; 2 Department of Reproductive and Genetics Medicine, Affiliated Hospital of Shandong University of Traditional Chinese Medicine, Jinan, Shandong, China

**Keywords:** progesterone, gonadotropin-releasing hormone antagonist protocol, fresh embryo transfer, normal ovarian response, live birth

## Abstract

**Objective:**

This study aimed to analyze the effect of progesterone (P) levels on the human chorionic gonadotropin (hCG) trigger day and the progesterone on hCG day-to-basal progesterone (P_hCG_/P_basal_) ratio on pregnancy outcomes in patients with a normal ovarian response undergoing fresh embryo transfer in a gonadotropin-releasing hormone antagonist (GnRH-ant) cycle.

**Materials and methods:**

This was a single-center retrospective study including 1,981 GnRH-ant protocol cycles with fresh embryo transfer from January 2017 to December 2023. All enrolled patients with a normal ovarian response were divided into four groups according to P levels on the hCG trigger day and P_hCG_/P_basal_ ratio based on cutoffs determined by receiver operating characteristic (ROC) analysis: Group A (P level<1.06 ng/mL and P_hCG_/P_basal_ ratio<1.2), Group B (P level <1.06 ng/mL and P_hCG_/P_basal_ ratio≥1.2), Group C (P level≥1.06 ng/mL and P_hCG_/P_basal_ ratio<1.2) and Group D (P level≥1.06 ng/mL and P_hCG_/P_basal_ ratio≥1.2). Subsequently, the associations between clinical variables and pregnancy outcomes were analyzed and compared across the groups.

**Results:**

The positive pregnancy rate (53.5%), clinical pregnancy rate (50.0%), live birth rate (LBR) (43.6%) and singleton rate (36.7%) were significantly higher in Group A than in the other three groups (all *P<0.001*). Furthermore, the LBR (30.2% vs. 22.4%) and singleton rate (23.8% vs. 17.1%) were significantly higher in Group C than in Group D (both *P<0.05*). The early miscarriage rate in Group A was comparable to Group B, but lower than in Group C and D (10.6% vs. 14.6% vs. 17.4% vs. 20.4%, *P = 0.016*). After multivariable logistic regression analysis, the LBR was highest in Group A (Group B vs.A: AOR = 0.437, 95% CI = 0.316-0.603, *P<0.001*; Group C vs.A: AOR = 0.512, 95% CI = 0.391-0.670, *P<0.001*; Group D vs.A: AOR = 0.325, 95% CI = 0.240-0.441, *P<0.001*).

**Conclusions:**

For patients with a normal ovarian response undergoing the GnRH-ant protocol, even a slight elevation in P levels on the hCG trigger day affected LBR. Moreover, the impact of the P_hCG_/P_basal_ ratio on pregnancy outcomes also warrants attention. A serum P level < 1.06 ng/mL on the hCG trigger day and P_hCG_/P_basal_ ratio < 1.2 were associated with a significantly higher LBR. When the P_hCG_/P_basal_ ratio ≥ 1.2, the LBR declined regardless of P levels on hCG trigger day. These findings highlight the potential for incorporating P_hCG_/P_basal_ ratio considerations into individualized clinical management strategies to optimize pregnancy outcomes.

## 1 Introduction

Progesterone (P) is a steroid hormone secreted primarily by luteinized granulosa and theca cells in the corpus luteum under the influence of luteinizing hormone (LH) during the second half of the menstrual cycle (1). Progesterone is essential for various critical processes, including embryonic development, implantation, and the maintenance of pregnancy. It is a critical regulator of endometrial receptivity, facilitates the transformation of endometrial stromal cells into decidual cells, and ensures embryonic survival (2). Established markers of endometrial receptivity, such as mucin-1 (MUC1), osteopontin, and integrins, are all regulated in a progesterone-dependent manner (3–6). In addition, successful embryo attachment requires a relatively quiescent uterine environment (7). Progesterone can reduce the expression of estrogen receptor-α (ERα), thereby further down-regulating oxytocin receptor levels, which are key mediators of uterine contractions (8). Progesterone also modulates intracellular calcium and potassium concentrations, prevents membrane potential changes, and inhibits actin–myosin crosslinking, collectively maintaining uterine smooth muscle quiescence (9–11). In most mammals, during the first two weeks of pregnancy, if the maternal immune system recognizes the embryo as a foreign entity, it may lead to pregnancy loss (12). Progesterone promotes the release of prostaglandin E2 (PGE2), a potent immunosuppressive factor, from fetal macrophages (13), and inhibits antigen presentation by dendritic cells, macrophages, and monocytes to T helper cells (14). Moreover, progesterone-induced blocking factor and galectins have been reported to further modulate maternal immune responses (15). In addition, progesterone influences uterine vascular formation and remodeling during pregnancy by affecting the expression of angiopoietin−2 (Ang−2) (16) and regulating VEGF−A–VEGFR2 signaling (17), which is critical for establishing adequate maternal–fetal blood flow and sustaining early embryonic development. Taken together, progesterone supports embryo implantation and pregnancy maintenance through multiple mechanisms, including regulating endometrial receptivity, suppressing uterine contractility, modulating maternal–fetal immune interactions, and influencing uterine vascular formation and remodeling.

In 1990, Edelstein et al. first reported the relationship between late-follicular-phase P levels and pregnancy outcomes in *in vitro* fertilization and embryo transfer (IVF-ET) cycles (18). Over the past 25 years, the impact of P levels on the pregnancy outcomes of IVF-ET has remained a central focus of research and academic interest. Elevated P levels during the late-follicular-phase was an independent risk factor that negatively impacts the clinical pregnancy rate (CPR) and live birth rate (LBR) in fresh IVF/intracytoplasmic sperm injection (ICSI) cycles (19). When the P levels surpass a certain threshold, the CPR and LBR after fresh ET decreased sharply (19). Premature elevation of P levels may lead to early luteinization, whereas delayed initiation could increase uterine contractions, which are negatively correlated with P dosage; increased uterine contractions on the day of ET can result in lower implantation rates (20). Although pituitary suppression with gonadotropin-releasing hormone (GnRH) analogues has dramatically reduced the incidence of premature LH surges during controlled ovarian hyperstimulation (COH) to 2% per cycle (21, 22), a slight elevation of late-follicular-phase P levels, not associated with premature LH surges, continues to occur in as many as 38% of all IVF cycles (23). Some studies have shown that elevated P levels are not detrimental to reproductive outcomes and may even be beneficial in certain patient groups (18, 24–26). In contrast, other studies have reported that low late-follicular-phase P levels can also negatively affect pregnancy outcomes (27, 28). The discrepancy in results between studies is mainly due to the uncertain threshold values for serum P levels. Most previous studies used absolute P levels on the day of human chorionic gonadotropin (hCG) administration, with the range typically from 0.8 ng/mL to 2.0 ng/mL (29). As a result, the predictive power of P levels alone has failed to demonstrate high sensitivity and specificity (30).

The possible mechanism underlying the increase in P levels which affects IVF outcomes include a higher number of mature follicles, excessive gonadotropin administration, increasingly high serum estradiol (E_2_) levels, aged oocytes and premature luteinization (29). As a result, more and more studies found ratio, such as the serum P/E_2_ ratio (31–34) and serum P/number of mature oocytes (35–37), as good predictors of assisted reproduction outcomes. Some studies indicated that basal P levels were the main and independent prognostic factor for P levels elevation at induction (38, 39). Mehmet et al. demonstrated that basal P levels are associated with increased preovulatory P levels rise but not with higher pregnancy rates in ICSI cycles with GnRH antagonists (GnRH-ant) (40). A single-center study of more than 10,000 cycles, investigated the effect of progesterone on hCG day-to-basal progesterone ratio (P_hCG_/P_basal_) on LBR in long agonist fresh IVF/ICSI cycles. It concluded P_hCG_/P_basal_ between 0.5-1.0 predicted a higher LBR in IVF (41).

The GnRH agonist (GnRH-a) protocol and GnRH-ant protocol are the most widely used protocols in COH cycles. Modest elevations in serum P levels can be detected at the end of the follicular phase, with incidence rates as high as 35% in GnRH-a regimens (18, 42)and 38% in GnRH-ant (43, 44). In the standard long GnRH-a protocol, steroid hormone levels are expected to be suppressed by lengthening GnRH suppression. This is not the case in standard GnRH-ant cycles in which pituitary suppression is not sustained during the early follicular phase. As a result, early follicular progesterone secretion might not be adequately suppressed due to inefficient luteolysis (45). GnRH-a improves the uniformity of follicle development (46). However, the prolonged duration of stimulation with follicle-stimulating hormone (FSH) increases the risk of ovarian hyperstimulation syndrome (OHSS) (47). GnRH-ant protocols with the advantages such as the shorter duration of the analog treatment, the shorter duration of stimulation with FSH and the lower risk of developing OHSS (48, 49), have been widely used in clinical practice since 2001, especially for normal and high ovarian responders (50, 51).

Individuals with a normal ovarian response generally have a low risk of OHSS and typically yield a stable number of retrieved oocytes and available embryos, making them ideal candidates for fresh ET (51). However, compared with the GnRH-a protocol, the GnRH-ant protocol is associated with a lower LBR per fresh ET cycle, although the cumulative LBR remains comparable (52). Therefore, this study aimed to analyze the effect of P levels on hCG trigger day and the P_hCG_/P_basal_ ratio on pregnancy outcomes in patients with a normal ovarian response undergoing the GnRH-ant protocol, in order to optimize pregnancy outcomes in fresh ET cycles.

## 2 Material and methods

### 2.1 Population and study design

We performed a retrospective, single-center cohort study at the Department of Reproduction and Genetics of the Affiliated Hospital of Shandong University of Traditional Chinese Medicine from January 2017 to December 2023. The study received approval from the Ethics Committee of the Affiliated Hospital of Shandong University of Traditional Chinese Medicine (Approval No (2025).101-KY) and conducted in accordance with relevant guidelines and regulations.

A total of 1,981 of GnRH-ant protocol cycles were included in the analysis, all involving the first IVF/ICSI cycle with fresh ET. This study included women with a normal ovarian response (50, 51, 53, 54): 1) aged 20–40 years; 2) 1.2 ng/mL≤anti-Müllerian hormone (AMH) < 4 ng/mL; 3) 6≤antral follicle count (AFC) < 16; 4) baseline FSH<10 IU/L; 5) 3< the number of oocytes retrieved <20. Exclusion criteria included: 1) polycystic ovary syndrome; 2) severe tubal hydrosalpinx or endometriosis; 3) untreated systemic or endocrine disorders such as diabetes mellitus and Cushing’s syndrome; 4) congenital uterine malformations or untreated endometrial pathology; 5) karyotypic abnormalities in either spouse; 6) male partner with azoospermia or genetic disorders.

### 2.2 Ovarian stimulation and laboratory protocols

All patients underwent a flexible GnRH-ant protocol. Briefly, from day 2 or 3 of the menstrual cycle, patients received daily injections of 150–225 IU gonadotropins (Gn), including recombinant FSH (rFSH, Gonal-f, Merck Serono, Switzerland or Puregon, MSD, USA) and/or urinary human menopausal gonadotropin (uHMG, Lizhu, China), with the dose adjusted according to individual characteristics such as age, body mass index (BMI), basal FSH levels, and AFC. Vaginal ultrasonography was performed, and serum LH and E_2_ levels were determined 3–5 days after the start of Gn injections. When the diameter of the dominant follicle reached 14 mm, or when it reached 12 mm accompanied by a serum E_2_ level reached 300 pg/mL, a daily dose of 0.25 mg GnRH antagonist (Cetrorelix, Merck, Darmstadt, Germany) was administered until the day of trigger. Following confirmation of the presence of ≥2 dominant follicles measuring ≥ 18 mm or ≥ 3 dominant follicles measuring ≥ 17 mm by ultrasound, in conjunction with P and E_2_ levels, final oocyte maturation was triggered by 4000–10000 IU human chorionic gonadotropin (hCG; Lizhu, China) or 250μg of recombinant human choriogonadotropin alfa solution (rhCG, Ovitrelle, Merck Serono, Germany). Oocyte retrieval was executed 34–36 hours after the trigger. A conventional IVF/ICSI approach was used for insemination, depending on the semen quality.

Luteal support was started on the day of oocyte retrieval with intramuscular injection of progesterone (20 mg, Zhejiang Xianju Pharmaceutical Co., China), at a daily dosage of 40 mg, and 30 mg oral dydrogesterone tablets daily (10 mg, Abbott Co., America) or progesterone vaginal sustained-release gel (90 mg, Merck Serono, Germany). The choice of luteal support regimen was individualized based on patient clinical characteristics, including endometrial thickness and serum progesterone levels, as well as the attending physician’s experience and patient preference. Embryo transfer was scheduled according to embryo quality, endometrial thickness, serum hormone profiles, and maternal age. One or two cleavage-stage embryos were transferred on day 3, or a single blastocyst was transferred on day 5 after oocyte retrieval. For cleavage-stage embryos (day 3), quality grading employed the Cummins criteria (55). Grade I and II embryos were classified as high quality and selected for fresh ET or vitrification. Suboptimal day 3 embryos (grades III-IV) were cultured to the blastocyst stage. Blastocyst quality was assessed according to Gardner’s scoring system (56) and embryos graded ≥3BB were categorized as high-quality blastocysts. High-quality embryos not selected for fresh ET were cryopreserved in a closed vitrification system. Serum β-hCG was measured 14 days after ET. A β-hCG level ≥ 25 IU/L was considered a positive pregnancy. Transvaginal ultrasonography was performed 3–4 weeks after ET to confirm intrauterine pregnancy. If an intrauterine pregnancy was confirmed, luteal support was continued until 55–60 days after ET.

### 2.3 Serum hormone levels measurement and grouping

Basal serum hormone levels, including FSH, LH, E_2_ and P, were measured between 7:00 and 11:00 a.m. using the automated Elecsys Immunoanalyzer (Beckman Coulter, Inc. USA) on day 2 of the cycle prior to exogenous gonadotropin stimulation. The intra-assay and inter-assay coefficients of variation were both less than 10%. On the hCG trigger day, venous blood was collected from patients between 7:00 and 9:00 a.m. And serum E_2_, LH and P levels were routinely assessed. If the serum P level exceeded 1.5 ng/mL, fresh ET was canceled and all embryos were cryopreserved in a closed vitrification system.

To determine the cutoff values of the serum P levels on the hCG trigger day and P_hCG_/P_basal_ ratio to discriminate between live birth and no live birth outcomes, a receiver operating characteristic (ROC) analysis was performed. The optimal thresholds—1.06 ng/mL for P levels and 1.2 for the P_hCG_/P_basal_ ratio—were identified based on the highest Youden index. Using these two ROC-derived cutoffs in combination, patients were further categorized into four groups in a 2×2 classification framework: Group A (P<1.06 ng/mL and P_hCG_/P_basal_ ratio<1.2), Group B (P<1.06 ng/mL and P_hCG_/P_basal_ ratio≥1.2), Group C (P≥1.06 ng/mL and P_hCG_/P_basal_ ratio<1.2) and Group D (P≥1.06 ng/mL and P_hCG_/P_basal_ ratio≥1.2). This combined grouping approach allowed assessment of the independent and joint predictive contributions of both progesterone indicators.

### 2.4 Outcome measures

The primary outcome of this study was the LBR, while secondary outcomes included the positive pregnancy rate, CPR, early miscarriage rate, and ectopic pregnancy rate. A positive pregnancy was defined as a serum β-hCG level≥25 IU/L measured 14 days after ET. Clinical pregnancy was defined as the ultrasonographic visualization of one or more gestational sacs, and included both intrauterine pregnancies and clinically documented ectopic pregnancies. Live birth was defined as the delivery of a viable neonate at or beyond 28 weeks of gestation. Early miscarriage was defined as a loss of clinical pregnancy before 12 weeks of gestation. Ectopic pregnancy, diagnosed by ultrasound, surgical visualization, or histopathology, refers to the implantation of a fertilized egg outside the uterine cavity.

### 2.5 Statistical analysis

Statistical analysis was performed using the Statistical Package for Social Sciences (SPSS) version 27.0 (SPSS Inc., Chicago, USA) and R4.4.3. Correlation analysis was performed using Spearman based on the R language. ROC analysis was performed to identify the most effective cutoff values of serum P levels on the hCG trigger day and the P_hCG_/P_basal_ ratio for discriminating between live birth and no live birth outcomes in women with a normal ovarian response undergoing the GnRH-ant protocol. The optimal cutoff values were determined according to the equivalent sensitivity and specificity and maximum value of the area under the ROC curve (AUC). Youden index (sensitivity + specificity-1) was used to choose the cutoff points of P level on the hCG day and P_hCG_/P_basal_ ratio. The AUC represents the probability of correctly predict the live birth.

Continuous variables adhering to a normal distribution were expressed as mean ± standard deviations (SD). The comparison of continuous variables between two groups was performed using Student’s *t*-test or Mann-Whitney U test. One-way ANOVA with *post-hoc* analysis or the Kruskal-Wallis rank-sum test was employed for comparisons between four groups. Chi-square (χ2) statistics or Fisher’s exact test was conducted to compare categorical variables between groups, with the results expressed as frequencies and percentages. Multivariable logistic regression analysis was utilized to assess the impact of known potential confounding factors on LBR in fresh ET cycles. These factors encompassed female age, BMI, infertility type (primary or secondary), basal serum FSH levels, method of laboratory insemination (IVF/ICSI), oocytes retrieved, endometrial thickness on the hCG trigger day, good-quality embryos (no/yes), number of embryos transferred (1/2) and type of transferred embryos (Cleavage embryo/Blastocyst). A *P*-value <0.05 was deemed indicative of statistical significance.

## 3 Results

### 3.1 Baseline and COH cycles characteristics of the patients

In this analysis, a total of 1981 cycles were evaluated. The general characteristics of women with and without live birth after fresh ET were shown in **Table 1**. Compared with the non-live birth group, women who achieved live birth were younger (*P* < 0.001), had a shorter duration of infertility (*P* = 0.012), lower basal FSH levels (*P* < 0.001), higher AMH levels *(P* < 0.001), more AFC (*P* < 0.001) and matured follicles (*P* < 0.001). The number of oocytes retrieved and embryos obtained were higher, while the total gonadotropin (Gn) dose used was lower in the live birth group (both *P* < 0.001). Furthermore, these women had lower serum P levels on the hCG trigger day, but a significantly thicker endometrial thickness compared to those without live birth (both *P* < 0.001). Additionally, a higher number of good-quality embryos were transferred in fresh ET cycles of women who achieved live birth (*P* < 0.001).

**Table 1:** Baseline and COH cycles characteristics of the patients.

Variables	Live birth (+)	Live birth (-)	P value
Number of patients	707	1274	
Age of woman (years)	32.60 ± 4.28	34.30 ± 4.47	**<0.001**
Infertility duration(years)	3.14 ± 1.97	3.38 ± 2.17	**0.012**
BMI (kg/m^2^)	24.15 ± 3.77	23.91 ± 3.40	0.160
Primary cause for infertility			0.584
Tubal factors	59.3% (419/707)	61.4% (782/1274)	
Male factors	5.4% (38/707)	5.5% (70/1274)	
Male+female factors	18.1% (128/707)	15.7% (200/1274)	
Unknown	17.3% (122/707)	17.4% (222/1274)	
Type of infertility			0.122
Primary infertility	40.7%(288/707)	37.2%(474/1274)	
Secondary infertility	59.3%(419/707)	62.8%(800/1274)	
Basal hormone level			
FSH (mIU/mL)	6.94 ± 1.42	7.28 ± 1.42	**<0.001**
LH (mIU/mL)	4.49 ± 1.69	4.38 ± 1.74	0.198
E_2_ (pg/ml)	37.36 ± 9.06	37.93 ± 9.12	0.178
P (ng/ml)	0.56 ± 0.30	0.55 ± 0.31	0.252
AMH (ng/ml)	2.43 ± 1.08	2.26 ± 0.98	**<0.001**
AFC	10.51 ± 3.31	9.87 ± 3.23	**<0.001**
Laboratory insemination			0.124
IVF	77.5%(548/707)	74.4%(948/1274)	
ICSI	22.5%(159/707)	25.6%(326/1274)	
Duration of stimulation (days)	8.99 ± 1.49	9.11 ± 1.75	0.118
Total dose of Gn (IU)	2149.40 ± 672.27	2274.23 ± 762.98	**<0.001**
Number of follicles ≥ 14 mm on hCG trigger day	9.00 ± 3.64	8.40 ± 3.52	**<0.001**
Oocytes retrieved	9.74 ± 3.74	9.09 ± 3.58	**<0.001**
Number of available embryos on day 3	3.69 ± 1.94	3.05 ± 1.75	**<0.001**
Good-quality embryos			**<0.001**
Yes	67.8%(479/707)	56.8%(723/1274)	
No	32.2%(228/707)	43.2%(551/1274)	
Endometrial thickness on the hCG trigger day (mm)	11.62 ± 2.05	10.99 ± 2.08	**<0.001**
Number of embryos transferred			0.178
Single	24.6%(174/707)	27.4%(349/1274)	
Double	75.4%(533/707)	72.6%(925/1274)	
Type of transferred embryos			0.327
Cleavage embryo	86.8% (614/707)	85.2% (1086/1274)	
Blastocyst	13.2% (93/707)	14.8% (188/1274)	
Hormone level on hCG day			
E_2_ (pg/ml)	2167.12 ± 890.86	2114.99 ± 901.61	0.216
P (ng/ml)	0.82 ± 0.35	0.97 ± 0.38	**<0.001**

Data are presented as mean ± SD for continuous variables and percentage (number/total) for categorical variables. Values in bold indicate statistical significance (*P*-value <0.05)

AFC, antral follicle count; AMH, anti-Müllerian hormone; BMI, body mass index; E_2_, estradiol; FSH, follicle stimulating hormone; Gn, gonadotropins; ICSI, intracytoplasmic sperm injection; hCG, human chorionic gonadotropin; IVF, *in vitro* fertilization; LH, luteinizing hormone; P, progesterone.

### 3.2 Correlation analysis

**Figure 1:**
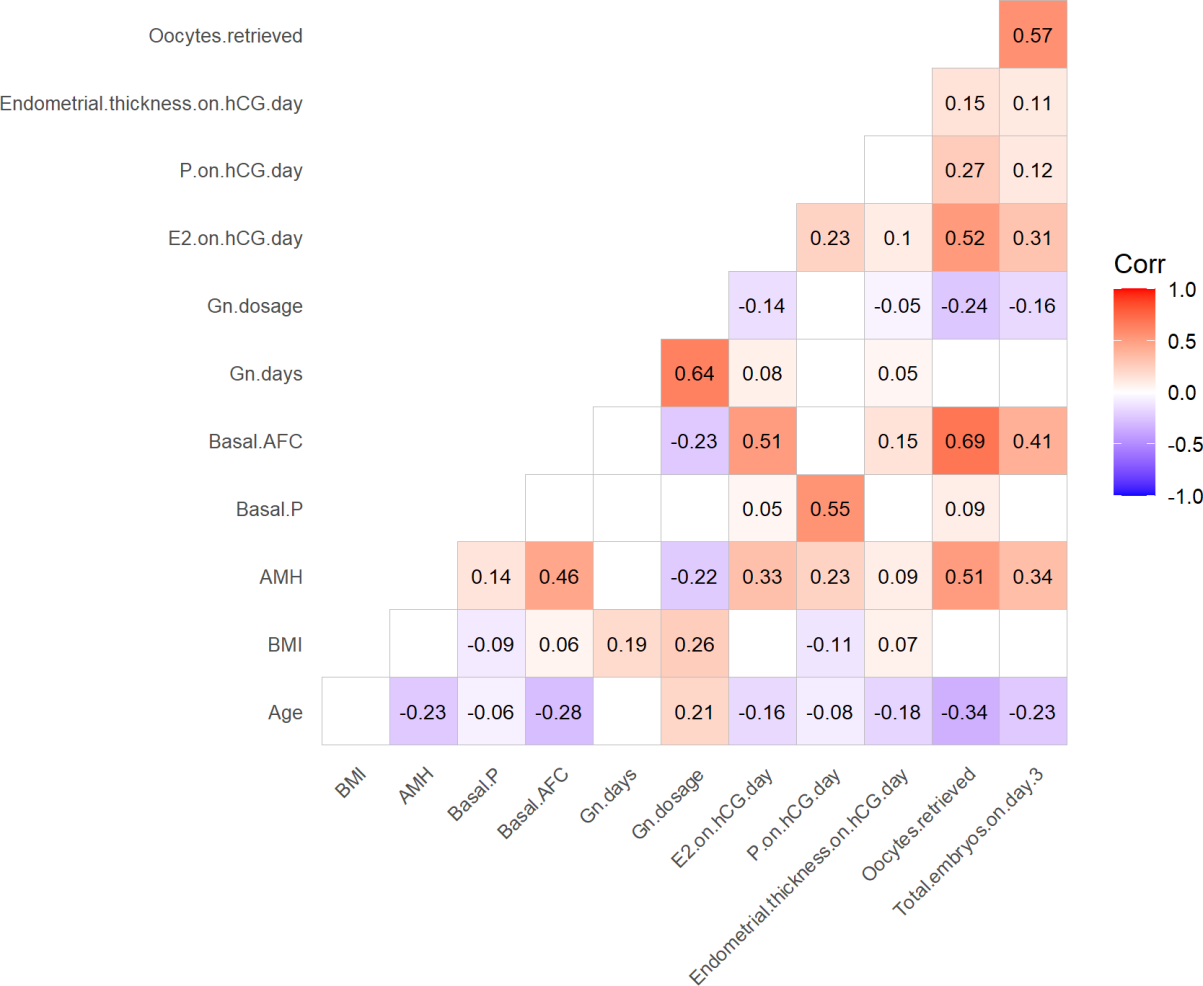
Correlation analysis between the P levels on the hCG trigger day and other indicators. AFC, antral follicle count; AMH, anti-Müllerian hormone; BMI, body mass index; E_2,_ estradiol; Gn, gonadotropins; P, progesterone.

**Figure 1** illustrates the results of the correlation analysis among the indicators. The P levels on the hCG trigger day showed a significant negative correlation with age (R= -0.08, *P =* 0.001) and BMI (R= -0.11, *P <*0.001). In contrast, significant positive correlations were observed between the P level on the hCG trigger day and AMH (R = 0.23, *P <*0.001), basal P level (R = 0.55, *P <*0.001), E_2_ level on the hCG trigger day (R = 0.23, *P <*0.001), number of oocytes retrieved (R = 0.27, *P<*0.001), as well as the number of embryos obtained (R = 0.12, *P <*0.001).

### 3.3 ROC results for P levels on the hCG trigger day and P_hCG_/P_basal_ ratio in predicting live birth

**Figure 2:**
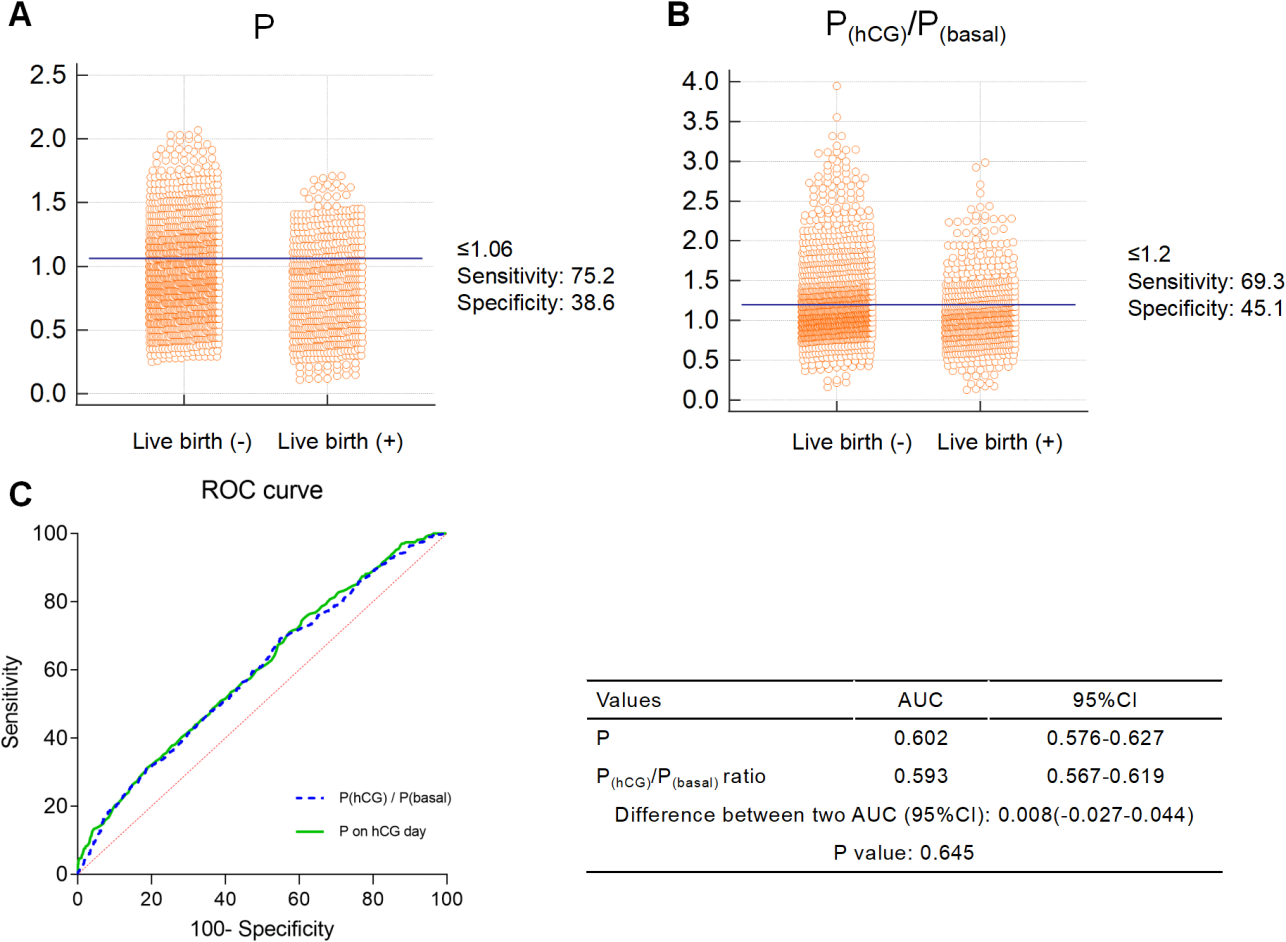
ROC curve. **(A)** Interactive dot diagram of P level on the hCG trigger day. The cutoff of P level is 1.06 ng/mL. **(B)** Interactive dot diagram of P_hCG_/P_basal_ ratio. The cutoff of P_hCG_/P_basal_ ratio is 1.2. **(C)** Comparison of ROC curves between P and P_hCG_/P_basal_ ratio based on live birth. AUC, area under the ROC curve; CI, confidence interval.

The ROC results are presented in **Figure 2**. The cutoff points of the P levels on the hCG trigger day and P_hCG_/P_basal_ ratio were determined using the Youden index. The cutoff value for serum P levels was 1.06 ng/mL (**Figure 2A**), and for the P_hCG_/P_basal_ ratio it was 1.2 (**Figure 2B**). The AUC for the P levels and P_hCG_/P_basal_ ratio in predicting live birth were 0.602 [95% confidence interval (CI): 0.576–0.627, *P <*0.001] and 0.593 (95% CI: 0.567–0.619, *P <*0.001), respectively. At the optimal cutoff value of 1.06 ng/mL, the P level showed higher sensitivity but lower specificity (75.2% and 38.6%, respectively). Similarly, the P_hCG_/P_basal_ ratio at the cutoff of 1.2 demonstrated higher sensitivity but lower specificity (69.3% and 45.1%, respectively). There was no statistically significant difference in the predictive performance between the P level and the P_hCG_/P_basal_ ratio for live birth, with an AUC difference of 0.008 (95% CI: − 0.027-0.044, *P* = 0.645) (**Figure 2C**).

### 3.4 Baseline and COH cycles characteristics of the patients with different levels of P and P_hCG_/P_basal_ ratio

According to the cutoff values of the P levels on the hCG trigger day and P_hCG_/P_basal_ ratio, patients were further divided into four groups. There were no statistically significant differences among the groups in terms of age, duration of infertility, BMI, type of infertility, infertility diagnosis, AMH, basal FSH, LH, E_2_, AFC, or method of laboratory insemination (all P>0.05). The total duration and dose of Gn use, endometrial thickness on the hCG trigger day, and the number, quality and type of embryos transferred were also comparable among the four groups (all P>0.05). However, significant differences were observed in the number of follicles ≥ 14 mm on the hCG trigger day, number of oocytes retrieved, and number of embryos obtained (all *P<*0.001), with Group D having the highest values and Group A the lowest. Similarly, E_2_ levels on the trigger day were highest in Group D and lowest in Group A, with the difference being statistically significant (*P<*0.001). Detailed data are shown in **Table 2**.

**Table 2:** Baseline and COH cycles characteristics of the patients with different levels of P and P_hCG_/P_basal_ ratio.

Variables	Group A	Group B	Group C	Group D	P value
Number of patients	1058	241	361	321	
Age of woman (years)	33.80 ± 4.46	33.72 ± 4.47	33.41 ± 4.60	33.63 ± 4.38	0.539
Infertility duration(years)	3.31 ± 2.15	3.43 ± 2.17	3.33 ± 2.05	3.12 ± 1.98	0.361
BMI (kg/m^2^)	24.22 ± 3.67	24.07 ± 3.50	23.76 ± 3.49	23.69 ± 3.06^a^	0.096
Primary cause for infertility					0.111
Tubal factors	59.3% (627/1058)	61.4% (148/241)	60.4% (218/361)	64.8% (208/321)	
Male factors	5.5% (58/1058)	5.4% (13/241)	6.1% (22/361)	4.7% (15/321)	
Male+female factors	15.3% (162/1058)	18.3% (44/241)	19.7% (71/361)	15.9% (51/321)	
Unknown	19.9% (211/1058)	14.9% (36/241)	13.9% (50/361)	14.6% (47/321)	
Type of infertility					0.761
Primary infertility	38.0% (402/1058)	39.0% (94/241)	40.7% (147/361)	37.1% (119/321)	
Secondary infertility	62.0% (656/1058)	61.0% (147/241)	59.3% (214/361)	62.9% (202/321)	
Basal hormone level					
FSH (mIU/mL)	7.22 ± 1.46	7.12 ± 1.37	7.13 ± 1.39	7.00 ± 1.39	0.091
LH (mIU/mL)	4.46 ± 1.67	4.40 ± 1.95	4.47 ± 1.66	4.42 ± 1.58	0.949
E_2_ (pg/ml)	37.30 ± 9.12	37.88 ± 9.01	38.01 ± 8.98	38.70 ± 9.2	0.094
P (ng/ml)	0.58 ± 0.29	0.27 ± 0.07^a^	0.84 ± 0.28^ab^	0.39 ± 0.12^abc^	**<0.001**
AMH (ng/ml)	2.29 ± 1.05	2.23 ± 0.82	2.37 ± 1.25	2.42 ± 0.81	0.086
AFC	10.12 ± 3.12	10.05 ± 3.39	10.21 ± 3.51	9.95 ± 3.4	0.760
Laboratory insemination					0.336
IVF	75.7% (801/1058)	73.9% (178/241)	73.1% (264/361)	78.8% (253/321)	
ICSI	24.3% (257/1058)	26.1% (63/241)	26.9% (97/361)	21.2% (68/321)	
Duration of stimulation (days)	9.00 ± 1.70	9.25 ± 1.53	9.16 ± 1.82	9.02 ± 1.45	0.102
Total dose of Gn (IU)	2218.41 ± 750.58	2230.43 ± 732.52	2282.03 ± 748.05	2207.41 ± 661.97	0.498
Number of follicles ≥ 14 mm on hCG trigger day	8.18 ± 3.49	8.81 ± 3.40^a^	8.80 ± 3.79^a^	9.69 ± 3.47^abc^	**<0.001**
Oocytes retrieved	8.66 ± 3.47	9.59 ± 3.49^a^	9.92 ± 3.83^a^	10.65 ± 3.64^abc^	**<0.001**
Number of available embryos on day 3	3.07 ± 1.75	3.43 ± 1.9^a^	3.34 ± 1.93^a^	3.54 ± 1.93^a^	**<0.001**
Good-quality embryos					0.894
Yes	60.9% (644/1058)	58.9% (142/241)	60.1% (217/361)	62% (199/321)	
No	39.1% (414/1058)	41.1% (99/241)	39.9% (144/361)	38% (122/321)	
Endometrial thickness on the hCG trigger day (mm)	11.30 ± 2.14	11.24 ± 1.97	11.08 ± 2.05	11.07 ± 2.07	0.193
Number of embryos transferred					0.144
Single	27.4% (290/1058)	27% (65/241)	27.7% (100/361)	21.2% (68/321)	
Double	72.6% (768/1058)	73% (176/241)	72.3% (261/361)	78.8% (253/321)	
Type of transferred embryos					0.320
Cleavage embryo	87.1% (921/1058)	83.8% (202/241)	83.7% (302/361)	85.7% (275/321)	
Blastocyst	12.9% (137/1058)	16.2% (39/241)	16.3% (59/361)	14.3% (46/321)	
Hormone level on hCG trigger day					
E_2_ (pg/ml)	1964.17 ± 750.21	2134.51 ± 840.01^a^	2261.29 ± 992.07^a^	2547.73 ± 1099^abc^	**<0.001**
P (ng/ml)	0.66 ± 0.22	0.84 ± 0.15^a^	1.30 ± 0.20^ab^	1.38 ± 0.23^abc^	**<0.001**
P (hCG)/P (basal)	0.72 ± 0.23	0.99 ± 0.39^a^	1.38 ± 0.44^ab^	1.55 ± 0.37^abc^	**<0.001**

Group A: P<1.06 ng/mL and P_hCG_/P_basal_ ratio<1.2; Group B: P<1.06 ng/mL and P_hCG_/P_basal_ ratio≥1.2; Group C: P≥1.06 ng/mL and P_hCG_/P_basal_ ratio<1.2; Group D: P≥1.06 ng/mL and P_hCG_/P_basal_ ratio≥1.2.

Data are presented as mean ± SD for continuous variables and percentage (number/total) for categorical variables. Values in bold indicate statistical significance (*P*-value <0.05).

AFC, antral follicle count; AMH, anti-Müllerian hormone; BMI, body mass index; E_2_, estradiol; FSH, follicle stimulating hormone; Gn, gonadotropins; ICSI, intracytoplasmic sperm injection; hCG: human chorionic gonadotropin; IVF, *in vitro* fertilization; LH, luteinizing hormone; P, progesterone.

a Compared with Group A, *P<0.05.*

b Compared with Group B, *P<0.05.*

c Compared with Group C, *P<0.05*.

### 3.5 Comparison of pregnancy outcomes with different levels of P and P_hCG_/P_basal_ ratio

**Table 3** presents the pregnancy outcomes for different levels of P and P_hCG_/P_basal_ ratio. The positive pregnancy rate (53.5%), CPR (50.0%), LBR (43.6%) and singletons rate (36.7%) were significantly higher in Group A than in the other three groups (all *P<*0.001). Furthermore, the LBR (30.2% vs. 22.4%) and singletons rate (23.8% vs. 17.1%) were significantly higher in Group C than in Group D (both *P<*0.05). The early miscarriage rate in Group A was comparable to Group B, but lower than in Group C and Group D (10.6% vs. 14.6% vs. 17.4% vs. 20.4%, *P = 0.016*). The ectopic pregnancy rate and twin rate were comparable among the four groups (P = 0.082 and P = 0.592, respectively).

**Table 3:** Pregnancy outcomes among the four groups.

	Group A	Group B	Group C	Group D	P value
Number of patients	1058	241	361	321	
Positive pregnancy rate	53.5% (566/1058)	43.2% (104/241)^a^	44% (159/361)^a^	40.5% (130/321)^a^	**<0.001**
Clinical pregnancy rate	50.0% (529/1058)	34.0% (82/241)^a^	38.2% (138/361)^a^	35.2% (113/321)^a^	**<0.001**
Ectopic pregnancy rate	1.9% (10/529)	3.7% (3/82)	3.6% (5/138)	6.2% (7/113)	0.082
Early miscarriage rate	10.6% (56/529)	14.6% (12/82)	17.4% (24/138)^a^	20.4% (23/113)^a^	**0.016**
Live birth rate	43.6% (461/1058)	27.0% (65/241)^a^	30.2% (109/361)^a^	22.4% (72/321)^ac^	**<0.001**
Singletons	36.7% (388/1058)	22.0% (53/241)^a^	23.8% (86/361)^a^	17.1% (55/321)^ac^	**<0.001**
Twins	6.9% (73/1058)	5.0% (12/241)	6.4% (23/361)	5.3% (17/321)	0.592

Group A: P<1.06 ng/mL and P_hCG_/P_basal_ ratio<1.2; Group B: P<1.06 ng/mL and P_hCG_/P_basal_ ratio≥1.2; Group C: P≥1.06 ng/mL and P_hCG_/P_basal_ ratio<1.2; Group D: P≥1.06 ng/mL and P_hCG_/P_basal_ ratio≥1.2.

Data are presented as percentage (number/total).

a Compared with Group A, *P<0.05* Compared with Group C, *P<0.05*. Values in bold indicate statistical significance (*P*-value <0.05).

**Figure 3:**
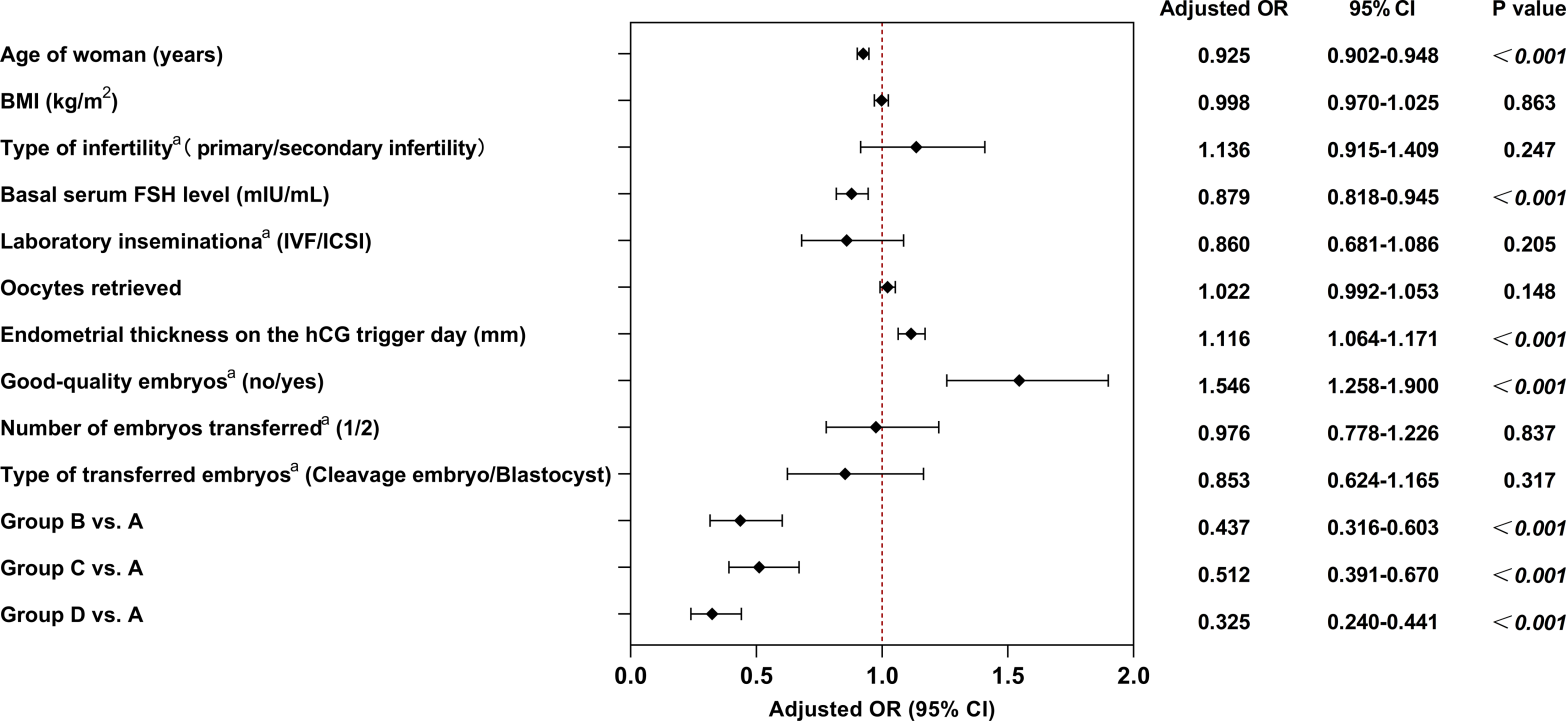
Multivariable logistic regression analysis with live birth as the influencing factor. ^a^denotes categorical variables with reference categories: primary infertility, IVF, no good-quality embryos, single embryo transfer, cleavage-stage embryos, and Group A. BMI, body mass index; FSH, follicle-stimulating hormone; ICSI, intracytoplasmic sperm injection; IVF, *in vitro* fertilization; hCG, human chorionic gonadotropin; OR, odds ratio; CI, confidence interval.

A multivariable logistic regression model was employed to adjust for potential confounding factors affecting LBR, including female age, BMI, infertility type (primary/secondary infertility), basal serum FSH level, laboratory insemination method (IVF/ICSI), number of oocytes retrieved, endometrial thickness on the hCG trigger day, presence of good-quality embryos (no/yes), number of embryos transferred (1/2), and type of transferred embryos (cleavage embryo/blastocyst). Multivariable logistic regression analysis revealed that female age (AOR = 0.925, 95% CI = 0.902-0.948, *P<*0.001), basal serum FSH level (AOR = 0.879, 95% CI = 0.818-0.945, *P<*0.001), endometrial thickness on the hCG trigger day (AOR = 1.116, 95% CI = 1.064-1.171, *P<*0.001), good-quality embryos (AOR = 1.546, 95% CI = 1.258-1.900, *P<*0.001) and different levels of P and P_hCG_/P_basal_ ratio were independent predictors of LBR. Regarding the P level and P_hCG_/P_basal_ ratio, the highest LBR was observed in Group A (P <1.06 ng/mL and P_hCG_/P_basal_ < 1.2). Compared to Group A, the adjusted odds ratios for LBR were significantly lower in all other groups: Group B vs.A: AOR = 0.437, 95% CI = 0.316-0.603, *P<*0.001; Group C vs.A: AOR = 0.512, 95% CI = 0.391-0.670, *P<*0.001; Group D vs.A: AOR = 0.325, 95% CI = 0.240-0.441, *P<*0.001. Detailed results are presented in **Figure 3**.

## 4 Discussion

To the best of our knowledge, this is the first study to explore the predictive value of P levels on the day of hCG administration and P_hCG_/P_basal_ ratio on pregnancy outcomes in fresh IVF/ICSI-ET cycles with a GnRH-ant protocol. In our study, correlation analysis revealed that higher P levels on the hCG day were associated with patients’ age, BMI, AMH, basal P levels and E_2_ levels on the hCG trigger day. In particular, basal P levels showed a significant positive correlation with P levels on the hCG trigger day (R = 0.55, *P<0.001*). While many previous studies have focused on evaluating the association between the P/E_2_ ratio on the hCG day and pregnancy outcomes in IVF/ICSI cycles (31, 34, 35, 57), studies examining the relationship between the P_hCG_/P_basal_ ratio on the hCG trigger day and pregnancy outcomes in normal ovarian responders undergoing fresh IVF/ICSI-ET cycles with a GnRH-ant protocol are lacking. A pilot study reported that higher basal P levels were negatively correlated with pregnancy outcomes in long agonist cycles without exploring the relationship between the basal P levels and the P levels on the hCG administration day (58). Enrico et al. demonstrated that basal P levels could be a major prognostic factor for elevated P levels on the hCG administration day (38). In the early follicular phase, basal serum P is primarily secreted by the adrenal glands (59), with a possible contribution from residual corpus luteum tissue due to incomplete luteolysis from the previous cycle (41, 60). At any time during a COH cycle, serum P levels reflect both adrenal and ovarian production (40). Therefore, women with elevated basal P levels are more likely to exhibit higher P levels on the hCG trigger day (39, 40).

In our study, the predictive values of the P_hCG_/P_basal_ ratio and P level on the hCG trigger day for live birth were evaluated using ROC curve analysis. The optimal cutoff value of P level on the hCG trigger day for predicting live birth was 1.06 ng/ml, while the cutoff value for the P_hCG_/P_basal_ ratio was 1.2. Many previous studies have used a threshold P level of 1.5–2.0 ng/mL on the hCG trigger day. However, the optimal cutoff value for predicting IVF outcomes has been shown to vary depending on ovarian response. According to the results of previous meta-analyses, a detrimental effect of progesterone elevation has been observed at levels as low as 0.8 to 1.1 ng/mL in both normal and poor responders. However, hyper-responders with a high oocyte yield (≥ 20 oocytes) showed negative effects only at P levels > 1.9 ng/ml on the hCG trigger day (61). In our study, we identified an optimal serum P cutoff value of 1.06 ng/mL on the hCG trigger day in women with normal ovarian response undergoing a GnRH-ant protocol, based on live birth outcomes. This finding is generally consistent with previous studies.

Patients were further divided into four groups according to the serum P level on the hCG trigger day and the P_hCG_/P_basal_ ratio, based on cutoff values determined by ROC analysis. Among the four groups, we observed that the group with low P level and a low P_hCG_/P_basal_ ratio (Group A) had the highest positive pregnancy rate, CPR, LBR and singletons rate, as well as the lowest early miscarriage rate. In pairwise comparisons, Group A (P level <1.06 ng/mL and P_hCG_/P_basal_ ratio <1.2) had a significantly higher positive pregnancy rate, CPR, LBR and singletons rate compared with Group B, C and D. The early miscarriage rate in Group A was significantly lower than that in Groups C and D. Furthermore, Group C (P level ≥1.06 ng/mL and P_hCG_/P_basal_ ratio <1.2) showed a significantly higher LBR compared with Group D. No statistically significant differences were observed in the remaining pairwise group comparisons. After adjusting for confounding factors using multivariable logistic regression analysis, Group A still showed a significantly higher LBR than Groups B, C, and D. Zhang et al. reported that among patients with normal ovarian response undergoing the GnRH-ant protocol, those with P ≥1.0 ng/mL had lower CPR and LBR compared to those with P <1.0 ng/mL. This finding is consistent with ours. However, in their study, the early miscarriage rate did not differ significantly between the two groups (51).

Mechanistic studies on the effects of elevated P on pregnancy outcomes have primarily focused on its impact on endometrial receptivity, as well as oocyte and embryo quality (62). While some studies suggest that higher P levels on the hCG trigger day impair embryo development (29, 63), most reports indicate minimal or no such effects (36, 64–66). For instance, a retrospective analysis of 68 oocyte donors and 68 recipients with ovarian failure clearly demonstrated that elevated P levels did not affect oocyte quality (65). Similarly, Neves et al. conducted a multicenter retrospective study of 1,495 ICSI cycles with preimplantation genetic testing for aneuploidy (PGT-A). Although the progesterone elevation group had significantly more euploid embryos and retrieved oocytes (both P = 0.001), there were no differences in euploid rates or blastocyst formation rates (64). Consistent with these findings, a prospective cohort study of 400 IVF/ICSI cycles also concluded that P levels had no negative impact on oocyte and embryo quality (67).These findings collectively imply that the reduced IVF success rates associated with high P levels are likely mediated by endometrial dysfunction rather than compromised oocyte or embryo quality. Human embryo implantation requires synchronized crosstalk between a receptive endometrium and a functional blastocyst, occurring within a narrow steroid-dependent “implantation window” (66). Evidence suggests that implantation rates decline significantly when embryo-endometrial asynchrony exceeds 3 ± 1.5 days (68). Elevated P levels may prematurely advance endometrial secretory transformation, thereby disrupting this synchronization (62). Supporting this, one study identified distinct endometrial gene expression profiles in patients with serum P >1.5 ng/mL on hCG day, potentially explaining the impaired receptivity and lower pregnancy rates observed (69). Animal studies further support this mechanism, as progesterone-treated mice showed fewer implantation sites, downregulated progesterone/estrogen receptors, and reduced expression of decidualization markers (70). In our study, the high progesterone group had more follicles >14 mm and retrieved oocytes, suggesting that excessive follicular recruitment contributed to the P elevation. Critically, there were no intergroup differences in the number/quality of transferred embryos, implying that the differences in final pregnancy outcomes were predominantly attributable to endometrial receptivity rather than embryonic factors.

In our study, the predictive ability of the P_hCG_/P_basal_ ratio (AUC = 0.593) for LBR was slightly lower than that of the P level on the hCG trigger day alone (AUC was 0.602), though the difference was not statistically significant (P = 0.645). One possible explanation is that basal P is primarily secreted by other organs such as the adrenal glands, and its levels tend to stabilize during controlled ovarian stimulation. In contrast, the P levels on the hCG trigger day reflect cumulative secretion from developing follicles. Therefore, we assume that the P_hCG_/P_basal_ ratio may diminish the prognostic value of the absolute P level on the hCG trigger day to some extent. Further analyses combining the P level on the hCG trigger day with the P_hCG_/P_basal_ ratio suggested that, regardless of whether the P level was elevated, LBR was reduced when the P_hCG_/P_basal_ ratio ≥1.2. A single-center retrospective study involving over 10,000 cycles found that a disproportionate changes between basal P levels and those on the hCG trigger day had an adverse effect on LBR in long GnRH agonist fresh IVF cycles. Notably, patients with a PhCG/Pbasal ratio between 0.5 and 1 exhibited significantly higher LBRs (41). However, that study did not further assess the predictive value of the P_hCG_/P_basal_ ratio in combination with the P level on the hCG trigger day for pregnancy outcomes.

In our reproductive center, fresh ET cycles were cancelled when serum P levels on the hCG trigger day exceeded 1.5 ng/mL, and whole embryo freezing was performed instead (23). However, the LBR in fresh ET cycles using the GnRH-ant protocol still remains lower than that observed in standard long GnRH-a protocols. Therefore, the present study was performed to explore the predictive value of serum P levels on the hCG trigger day for pregnancy outcomes in fresh IVF cycles with GnRH-ant regimen. We acknowledge that the retrospective and single-center cohort nature of our study poses certain limitations. Nevertheless, the single-center nature also ensured a homogeneous patient population. In addition, we minimized the influence of confounding factors by including only patients with normal ovarian responses undergoing the GnRH-ant protocol. Serum P level measurements were routinely conducted by trained professionals in our central laboratory, ensuring high reliability. Another strength of the study is its relatively large sample size and complete follow-up data. Our findings require further confirmation in high-quality randomized controlled trials. Future studies should also investigate similar associations in populations with high or poor ovarian responses and analyze the impact of the quality of different embryos or blastocysts transferred on pregnancy outcomes.

## 5 Conclusion

For patients with a normal ovarian response undergoing the GnRH-ant protocol, even a slight elevation in P levels on the hCG trigger day adversely affected the LBR. Moreover, the impact of the P_hCG_/P_basal_ ratio on pregnancy outcomes also warrants attention. A serum P level <1.06 ng/mL on the hCG trigger day combined with a P_hCG_/P_basal_ ratio <1.2 was associated with a significantly higher LBR. When the P_hCG_/P_basal_ ratio was ≥1.2, the LBR declined regardless of the P levels on the hCG trigger day. These findings highlight the potential value of incorporating the P_hCG_/P_basal_ ratio into individualized clinical management strategies to optimize pregnancy outcomes.

## Data Availability

The original contributions presented in the study are included in the article/supplementary material. Further inquiries can be directed to the corresponding authors.
